# Mucin‐producing urothelial‐type adenocarcinoma of the prostate diagnosed after robot‐assisted radical prostatectomy

**DOI:** 10.1002/iju5.12380

**Published:** 2021-09-25

**Authors:** Kosuke Shimizu, Ryo Inoue, Shinobu Tomochika, Naohito Isoyama, Yoshiaki Yamamoto, Hiroaki Matsumoto, Koji Shiraishi, Shigefumi Yoshino, Toyonori Tsuzuki, Hideyasu Matsuyama

**Affiliations:** ^1^ Departments of Urology Graduate School of Medicine Yamaguchi University Ube Japan; ^2^ Department of Gastroenterological Breast and Endocrine Surgery Graduate School of Medicine Yamaguchi University Ube Japan; ^3^ Departments of Surgery Kanmon Medical Center Shimonoseki Japan; ^4^ Department of Surgical Pathology School of Medicine Aichi Medical University Nagakute Japan

**Keywords:** immunohistochemistry, mucinous adenocarcinoma, prostate, urethra, urothelial‐type

## Abstract

**Introduction:**

Mucin‐producing adenocarcinoma of the prostate is a rare disease that includes prostate adenocarcinoma with mucus production, secondary adenocarcinoma from the bladder or colorectum, and adenocarcinoma from the urothelium of the prostatic urethra. We describe prostate‐specific antigen‐negative mucin‐producing urothelial‐type adenocarcinoma of the prostate.

**Case presentation:**

The patient had urinary retention and a serum prostate‐specific antigen level of 0.74 ng/mL. Computed tomography and magnetic resonance imaging revealed a prostate tumor with a mucous component. We diagnosed adenocarcinoma by prostate biopsy and subsequently performed robot‐assisted radical prostatectomy. Mucin‐producing urothelial‐type adenocarcinoma of the prostate was diagnosed by pathological examinations. Lung metastasis, developing within 3 months after surgery, was treated using chemotherapy.

**Conclusion:**

Endocrine therapy is ineffective for mucin‐producing urothelial‐type adenocarcinoma of the prostate. Mucin‐producing urothelial‐type adenocarcinoma of the prostate diagnosis requires pathological and immunohistochemical analyses. It is important to surgically remove the primary lesion, and robot‐assisted radical prostatectomy may provide an effective approach. Multimodal therapy is essential to treat for mucin‐producing urothelial‐type adenocarcinoma of the prostate.

**Abbreviations & Acronyms**
CT: computed tomography
FDG: fluorodeoxyglucose
MPUAP: mucin‐producing urothelial‐type adenocarcinoma of the prostate
MRI: magnetic resonance imaging
PET: positron emission tomography
PSA: prostate‐specific antigen
RARP: robot‐assisted radical prostatectomy

**Keynote message**

PSA‐negative MPUAP is difficult to diagnose using a prostate biopsy. In localized imaging‐suspect, mucin‐producing prostate tumors, even if PSA negative, surgical intervention is imperative.

## Introduction

MPUAP is a rare entity, and only 25 cases have been reported in the literature. Similar diseases include mucinous adenocarcinoma of the prostate and mucin‐producing secondary adenocarcinoma originating in the bladder or colorectal tissues. However, patients with MPUAP may have a poor prognosis compared with typical prostate cancer, and androgen deprivation therapy is ineffective for MPUAP. Radical prostatectomy may be a feasible treatment when clinically localized. Whereas prognosis will be poor in patients with metastasis, since no evidenced chemotherapy regimen exists. Here, we report a case of PSA‐negative MPUAP.

## Case presentation

**Fig. 1:**
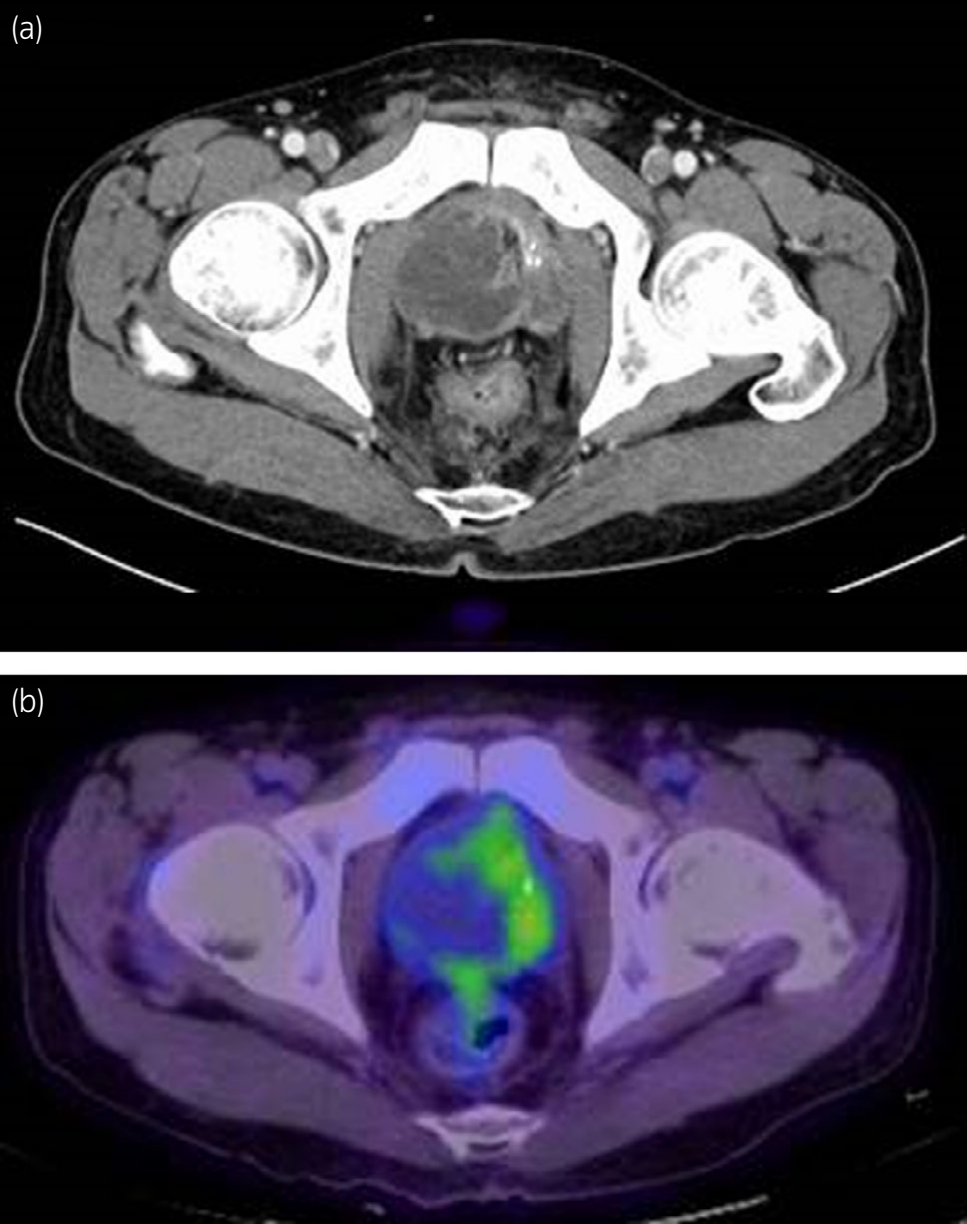
Contrast‐enhanced CT and PET‐CT images of the pelvis at diagnosis. (a) Transverse section, CT: A mucus‐enriched prostate tumor was detected with slight contrast at the edge. (b) Transverse section, PET‐CT: The prostate tumor showed an accumulation of FDG at the contrast‐enhanced site of the CT image.

**Fig. 2:**
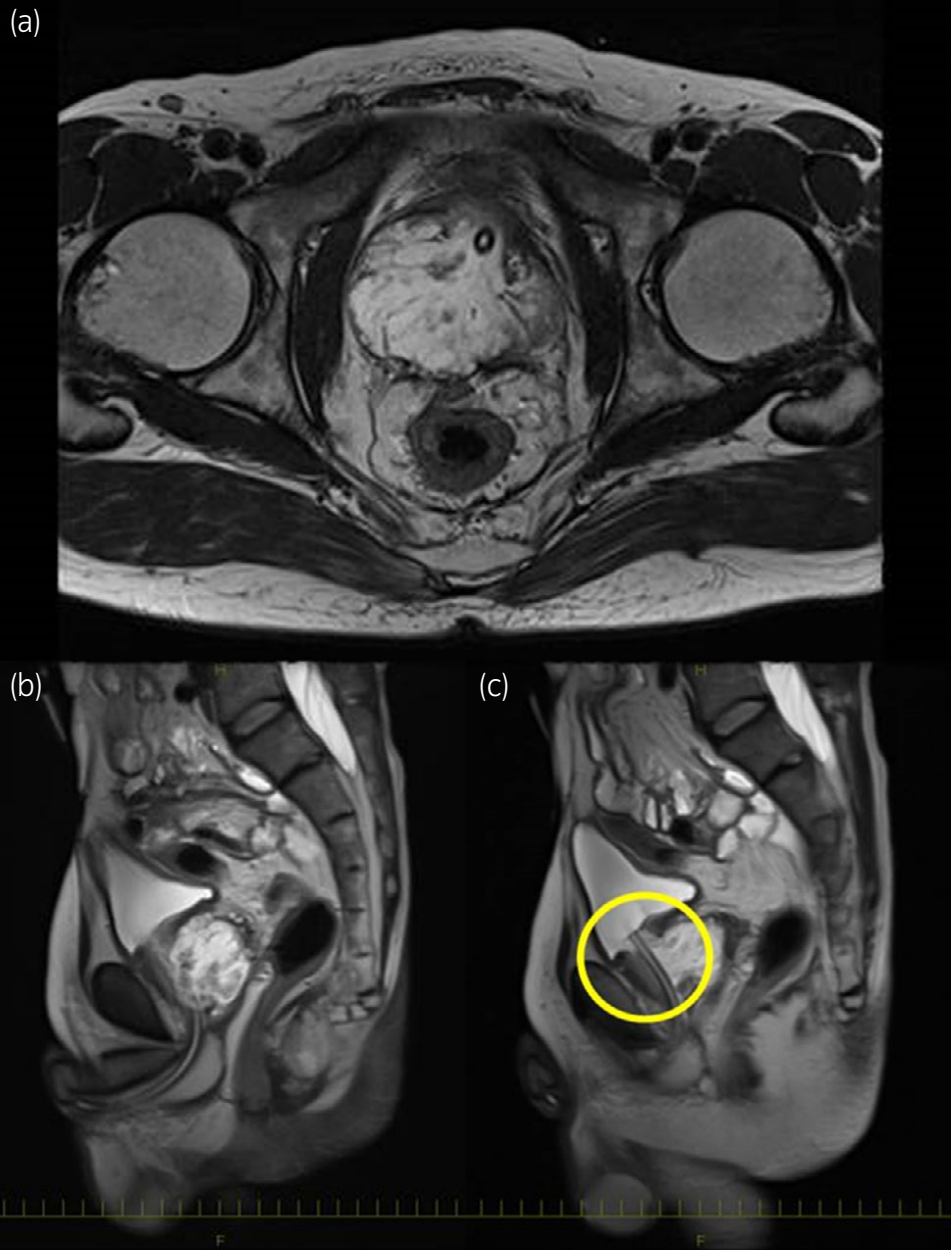
MRI of the pelvis at diagnosis. (a,b) Transverse and sagittal sections of T2‐weighted MRI: A mucus‐enriched prostate tumor was detected, similar to the contrast‐enhanced CT and PET‐CT images. (c) Sagittal section of T2‐weighted MRI suggesting that a mucinous tumor of the prostate extended to the muscle of the bladder (inner yellow circle).

A 59‐year‐old male visited our department for the treatment of ischuria. He had a history of surgery for an imperforate anus in childhood, following multiple ileus operations. A digital rectal examination of the prostate revealed a stony‐hard with an irregular surface. Ultrasonography showed an enlarged prostate (estimated volume; 83 cm^3^), and the serum PSA level was 0.74 ng/mL. CT revealed a mucus‐enriched prostate tumor with slight contrast at the edge (Fig. 1a). PET‐CT showed the accumulation of FDG at the same site (Fig. 1b). MRI revealed a prostate tumor with fluid retention, suggesting the possibility of invasion of the bladder and rectum (Fig. 2a–c). We suspected the possibility of bladder infiltration on cystoscopy but not of rectal infiltration on colonoscopy. No radiographic metastasis was noted (cT3aN0M0).

Transrectal prostate biopsies showed adenocarcinoma of unknown primary origin. We planned to perform a total cystoprostatectomy. However, we decided to perform RARP based on the intraoperative findings. There was a severe desmoplastic reaction between prostate and rectum. Although the rectal muscle was visually intact, an ileostomy was performed by the gastroenterological surgeon.

**Fig. 3:**
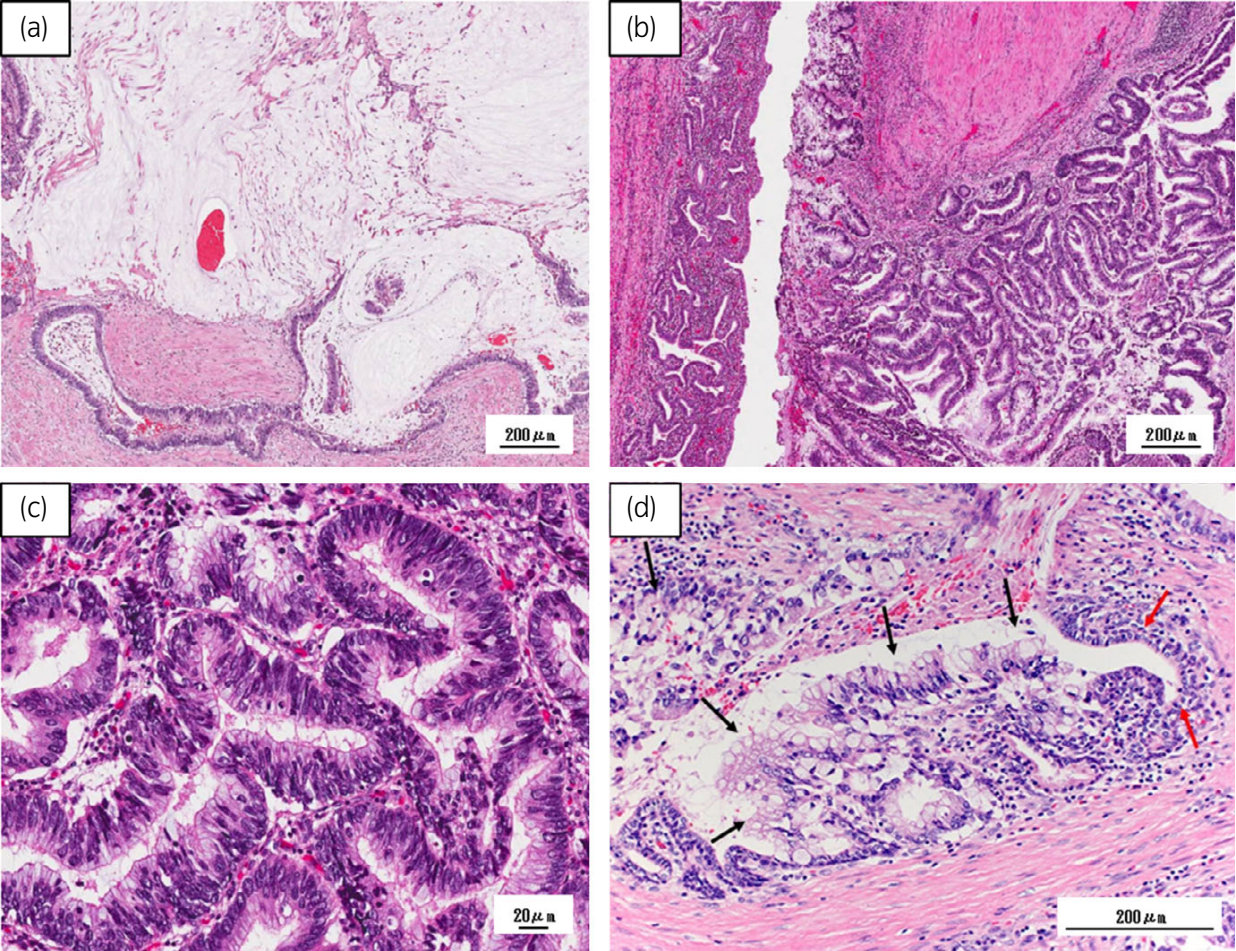
Histological analysis of the surgically excised prostate. (a) There was a rich accumulation of mucus inside the tumor. (b) The urethral mucosa on the right side was invaded by adenocarcinoma cells, in contrast to the intact mucosa on the left side. (c) Tumor cells with mucus in the cytoplasm formed gland ducts with characteristics histologically similar to those of colorectal adenocarcinoma. (d) Tumor cells invading the normal urothelium (black arrow, tumor cells: red arrow, urothelium).

**Fig. 4:**
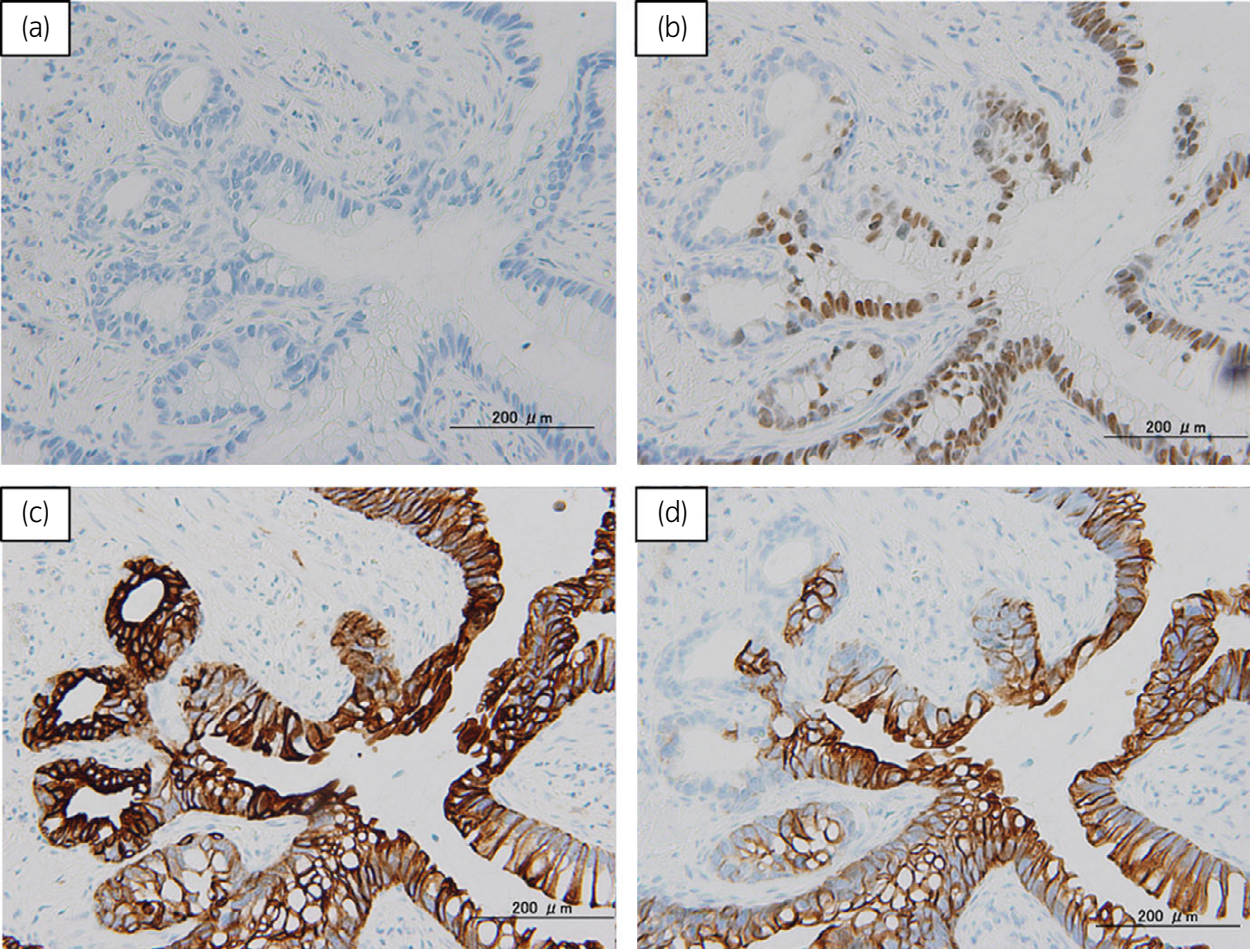
Immunohistochemical analysis of the surgically excised prostate. (a) PSA was not detected in tumor cells. (b) CDX2 was sparsely positive in tumor cells. (c) CK7 was highly positive in tumor cells. (d) CK20 was sparsely positive in tumor cells.

Total surgical time was 8 h and 49 min with a blood loss of 315 mL. Histological examination revealed adenocarcinoma originating in the prostatic urethra (Fig. 3a–d). The tumor produced a rich accumulation of mucus (Fig. 3a) and the urethral mucosa was invaded by adenocarcinoma cells (Fig. 3b,d). Tumor cells formed glands with characteristics histologically similar to those of colorectal adenocarcinoma (Fig. 3c). PSA‐negative MPUAP was diagnosed by immunohistochemistry (pT3bN0M0, Fig. 4a–d). After surgery, the patient experienced no complication other than controllable urinary incontinence, and was discharged 18 days after surgery. Lung metastasis was noted 3 months postoperatively. Concerning treatment of metastases, we consulted with gastroenterological doctor and anticancer chemotherapy (capecitabine plus oxaliplatin [XELOX]) was selected according to the adenocarcinoma (colon cancer) regimen. Due to lung metastasis progression (growth of lung metastatic lesions) after 6 months, we administered capecitabine plus bevacizumab as second‐line chemotherapy. However, local recurrence appeared in the pelvis 3 months later. Subsequently, we performed total pelvic exenteration and stable disease was followed by 2 months without de novo metastatic lesion.

## Discussion

Mucinous adenocarcinoma of the prostate is very rare, especially MPUAP, as indicated by 25 cases reported in the English literature.

Twenty‐two of the 25 patients had voiding difficulty, and interestingly, seven experienced the characteristic symptom of mucusuria. The present case had voiding difficulty as a chief complaint, while no mucusuria. As for the treatment in the literature, transurethral resection of the prostate was selected in 12 patients, although 11 died at an average of 40.5 months from the presentation. The most frequent site of metastasis was the lung in 5 cases, followed by the liver, bone, and pelvic wall (3, 2 and 2 cases, respectively).

Although prostate primary mucinous adenocarcinoma has better a prognosis than adenocarcinoma of the prostate,^1^ the prognosis of MPUAP may be much worse than prostate primary mucinous adenocarcinoma. Because of the fact that endocrine therapy is ineffective for MPUAP from urothelium origin with no PSA expression, it is very important to differentiate PSA‐positive adenocarcinoma from PSA‐negative primary prostate mucinous adenocarcinomas.^2^

Previous reports have indicated that MPUAP originates from urethral glanditis with malignant transformation or prostatic urethral dysplasia. Histological features are similar between MPUAP and bladder adenocarcinoma, both of which are reportedly caused by inflammation. It is, therefore, important to exclude bladder adenocarcinoma to make a correct diagnosis.^3^, ^4^, ^5^

Although serum CA19‐9 is reportedly high in MPUAP,^6^, ^7^ no definite tumor marker exists. Immunohistochemical analysis is useful for the diagnosis of MPUAP,^3^, ^8^, ^9^, ^10^ suggesting the need for the surgical excision of the primary lesion.^3^, ^4^ MPUAP is negative for PSA or CDX2 (which is frequently positive in cases with enteric features),^4^ but positive for CK7, CK20, CK34βE12 (potential urothelial markers), and CEA in immunohistochemical analysis.^11^ The present case revealed negative PSA and positive CDX2, CK7, and CK20 findings. Although this case could be similar to enteric features because of positive CDX2, it was finally diagnosed as MPUAP by pathological features (negative PSA, positive CK7 and CK20).

To the best of our knowledge, this is the first case that MPUAP was treated with RARP. However, lung metastases appeared only 3 months postoperatively. Radiotherapy, chemotherapy, and chemoradiation therapy has been reported as a treatment for metastatic lesion.^3^, ^12^ We chose chemotherapy to manage metastases. Past two cases were treated by chemotherapy including oral capecitabine.^3^, ^12^ We selected XELOX therapy according to the regimen with unresectable advanced recurrent colorectal cancer because the histopathology of the tumor's glandular structure was similar to that of colon cancer (Fig. 3c). Capecitabine is fluoropyrimidine, a prodrug of 5‐FU, that is converted to a precursor in the liver, selectively metabolized in tumor tissue, and then converted to 5‐FU.^13^ Although 5‐FU is mainly used to treat gastric, rectal, and breast adenocarcinomas, it is also used to treat mucinous adenocarcinoma of the bladder.^14^ Although its effectiveness is unknown, our patient’s cancer did not progress for at least 5 months after chemotherapy and the effect can be expected to prevent the progression of MPUAP. However, it is considered to be necessary to perform multimodal therapy as in this case, because MPUAP is a disease with a poor prognosis. Further strict follow‐up is required.

## Conclusion

To the best of our knowledge, this is the first report of surgical removal of MPUAP by RARP. Since MPUAP has a different biological feature from adenocarcinoma of the prostate, correct diagnosis by immunohistochemical analysis and surgical removal of the primary organ, if possible, is mandatory both for the correct diagnosis and treatment.

## Conflict of interest

The authors declare no conflict of interest.

## Approval of the research protocol by an Institutional Reviewer Board

Not applicable.

## Informed consent

Not applicable.

## Registry and the Registration No. of the study/trial

Not applicable.
